# Molecular basis for the distinct divalent cation requirement in the uridylylation of the signal transduction proteins GlnJ and GlnB from *Rhodospirillum rubrum*

**DOI:** 10.1186/1471-2180-12-136

**Published:** 2012-07-08

**Authors:** Pedro Filipe Teixeira, Maria A Dominguez-Martin, Stefan Nordlund

**Affiliations:** 1 Department of Biochemistry and Biophysics, Stockholm University, Stockholm, SE-106 91, Sweden

**Keywords:** PII proteins, Post-translational modification, Uridylyltransferase

## Abstract

**Background:**

PII proteins have a fundamental role in the control of nitrogen metabolism in bacteria, through interactions with different PII targets, controlled by metabolite binding and post-translational modification, uridylylation in most organisms. In the photosynthetic bacterium *Rhodospirillum rubrum*, the PII proteins GlnB and GlnJ were shown, in spite of their high degree of similarity, to have different requirements for post-translational uridylylation, with respect to the divalent cations, Mg^2+^ and Mn^2+^.

**Results:**

Given the importance of uridylylation in the functional interactions of PII proteins, we have hypothesized that the difference in the divalent cation requirement for the uridylylation is related to efficient binding of Mg/Mn-ATP to the PII proteins. We concluded that the amino acids at positions 42 and 85 in GlnJ and GlnB (in the vicinity of the ATP binding site) influence the divalent cation requirement for uridylylation catalyzed by GlnD.

**Conclusions:**

Efficient binding of Mg/Mn-ATP to the PII proteins is required for uridylylation by GlnD. Our results show that by simply exchanging two amino acid residues, we could modulate the divalent cation requirement in the uridylylation of GlnJ and GlnB.

Considering that post-translational uridylylation of PII proteins modulates their signaling properties, a different requirement for divalent cations in the modification of GlnB and GlnJ adds an extra regulatory layer to the already intricate control of PII function.

## Background

Members of the PII family of signal transduction proteins are fundamental molecular messengers involved in the regulation of nitrogen metabolism in bacteria, archaea and eukarya (plants) [1,2]. These proteins exert their role at different levels: they regulate gene expression by modulating the activity of several transcription factors [3], they control the flux through an ammonium transport protein [4] and influence the activity of key metabolic enzymes, e.g. glutamine synthetase (GS) and nitrogenase [5,6].

PII proteins are trimers of about 37 kDa, with each monomer containing a double βαβ ferredoxin fold. It has been previously shown that each trimer can bind up to three molecules of 2-oxoglutarate (2-OG) and ATP/ADP allowing the sensing of the carbon/nitrogen and energy status in the cell [7,8]. In the different structures of PII proteins solved so far, one of the most striking characteristics is the existence of three surface exposed loops per monomer, the B, C and T-loops [2]. The three nucleotide-binding sites (where ATP and ADP bind) are located in the inter-subunit clefts formed by the interaction of the B and C loops. The binding of ATP displays negative cooperativity (as does 2-OG binding), with ADP competing for the same binding site, as was shown for GlnB from *Escherichia coli *[7]. Recent structures of *Synechococcos elongatus* GlnB and *Azospirillum brasilense* GlnZ have convincingly elucidated the 2-OG binding sites within PII proteins and established that this binding influences protein conformation, particularly of the T-loop region [9,10]. Moreover, the structure of *S. elongatus* GlnB also provided an explanation for the negative cooperativity observed in the binding of 2-OG, considering that binding of the first 2-OG molecule generates unequal binding sites in the other two subunits [9].

In most proteobacteria, including the photosynthetic nitrogen-fixing bacterium *Rhodospirillum rubrum*, PII proteins are covalently modified by reversible uridylylation at tyrosine 51 in the T-loop, yielding 0–3 subunits modified with UMP per trimer. The uridylyltransferase and uridylylremoving activities are catalyzed by the bifunctional enzyme uridylyltransferase GlnD, with the reactions being regulated by the concentration of 2-oxoglutarate, through binding to the PII proteins [11]. The two activities of *R. rubrum* GlnD occur at distinct active sites, with the N-terminal nucleotidyltransferase domain involved in PII uridylylation and the central HD domain responsible for PII-UMP deuridylylation [12].

In *R. rubrum*, three PII proteins have been identified and named GlnB, GlnJ and GlnK [6]. However, only GlnB and GlnJ have been extensively studied and found to have both unique and overlapping functions in the regulation of gene transcription (two-component system NtrBC), ammonium transport (AmtB) and activity of metabolic enzymes GS and nitrogenase (by regulating the DRAT/DRAG system). While both proteins can regulate the activity of the adenylyltransferase GlnE (and thereby controling GS activity), GlnB specifically regulates NtrB and DRAT and GlnJ has a preferential role in the regulation of AmtB and possibly DRAG [5,6,13-15].

Even though GlnB and GlnJ share 68% sequence identity, the conditions for in vitro uridylylation by GlnD are different [11]. In the uridylylation assays with purified *R. rubrum* GlnD and PII proteins, efficient uridylylation requires the presence of ATP, 2-OG and a divalent cation. However, the uridylylation of GlnJ only occurred when Mn^2+^ was present, while the uridylylation of GlnB occurred with either Mg^2+^ or Mn^2+^[11]. Although the structure of neither of the *R. rubrum* PII proteins has been determined, it is possible that their T-loop assumes different conformations, by analogy with the recent structural data from PII proteins from *A. brasilense* and *S. elongatus *[9,10]. Considering these probably different conformations, it can be hypothesized that the correct conformation of the T-loop in GlnJ required for the interaction with GlnD is only achieved in the presence of Mn^2+^ (or Mn-ATP).

Considering that these differences in the divalent cation required to promote uridylylation of the PII proteins might be of functional significance, we have aimed at elucidating the molecular basis for this difference.

## Results and discussion

### Preliminary considerations

It was previously shown that the in vitro uridylylation of GlnJ catalyzed by purified GlnD requires the presence of Mn^2+^ ions, in addition to ATP and 2-OG [11]. Moreover, this functional connection between GlnJ and Mn^2+^ is supported by additional studies. We have shown that dissociation of the complex formed between GlnJ and the membrane embedded ammonium transport protein AmtB1 is favored by 2-OG and ATP but only in the presence of Mn^2+^[13]. Also, in the same study it was observed that the uridylylation of endogenous *R. rubrum* GlnJ recovered from the membrane fraction was only possible in the presence of Mn^2+^. In contrast to GlnJ, GlnB was efficiently uridylylated in the presence of either Mg^2+^ or Mn^2+^[11].

Although GlnD itself is known to bind both Mg^2+^ and Mn^2+^[16], the fact that uridylylation of GlnB occurs with either of the divalent cations present lead us to hypothesize that the reason for the different response to divalent cations in the uridylylation of GlnB and GlnJ is related to the PII protein and not to GlnD itself. Based on this premise we assumed that exchanging specific amino acid residues in GlnJ to the ones in GlnB might enable GlnJ to also be modified in the presence of Mg^2+^ as the only cation present.

The deuridylylation of both GlnB-UMP and GlnJ-UMP (also catalyzed by GlnD) was shown previously to require Mn^2+^, but Mg^2+^ did not support this activity in the *R. rubrum* enzyme [11], in contrast to *E. coli* GlnD [16].

### Sequence analysis

**Figure 1:**
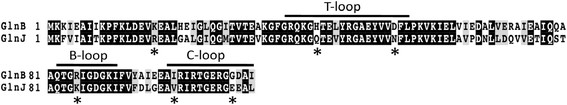
** Alignment of the amino acid sequence of the *R. rubrum *GlnB and GlnJ proteins, constructed using ClustalW** (http://www.ebi.ac.uk/Tools/clustalw2/index.html). The loop regions are highlighted and the positions of the amino acid substitutions used in this study are marked with a star.

The *R. rubrum* GlnB and GlnJ proteins are composed of 112 amino acids with 68% sequence identity. Figure 1 represents an alignment of the amino acid sequences of GlnB and GlnJ. In this alignment it is clear that these proteins contain large stretches of almost completely conserved residues, alternating with regions with lower conservation. We have focused on the regions of higher conservation, hypothesizing that even small differences in these areas might have an important structural/functional effect. Using this criterion we constructed GlnJ variants with the following substitutions: R17K, Q42H, N54D, K85R, V100M and E109G (in each position the residue in GlnJ was replaced by the corresponding one in GlnB). These variants were expressed and purified as N-terminal histidine tagged fusions.

Although not all the residues selected are located in regions of the PII protein that have previously been shown to be involved in metabolite binding, we decided to analyze amino acids occurring in areas of high conservation as, due to the considerable flexibility of the PII structure, they may also play a role in this response to divalent cations. An example of this high flexibility comes from the recent structure of *S. elongatus* GlnB, where the very C-terminal portion of the protein displays a large conformational change upon binding of the ligands to the T-loop region [9].

### Uridylylation of GlnJ variants in the presence of Mn^2+^ and Mg^2+^

Using purified GlnD and GlnJ variants we analysed the uridylylation profile in the conditions that were previously determined [11] and described in the Materials and methods, with either Mg^2+^ or Mn^2+^ present in the assays.

**Figure 2:**
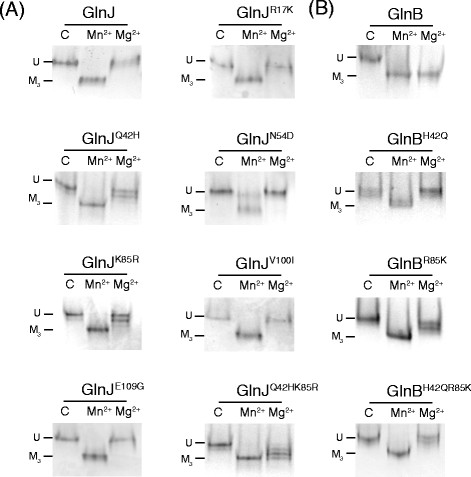
** Uridylylation of GlnJ (A) and GlnB (B) variants.** The reactions were performed as described in the Materials and methods in the presence of Mn^2+^, Mg^2+^ or without either divalent cation (control - C), and the uridylylation status analyzed by native PAGE. U – unmodified, M3- modified (fully modified trimmers).

As shown in Figure 2, GlnJ is only extensively modified in the presence of Mn^2+^ (A) while GlnB is modified with both Mn^2+^ and Mg^2+^ (B), as analyzed by native PAGE, with a slower migrating band converted to a faster migrating band (all 3 subunits modified). The identity (and uridylylation status) of the two forms was also confirmed by mass spectrometry (results not shown). The GlnJ variants R17K, V100I and E109G showed the same pattern as GlnJ (Figure 2A). The GlnJ^N54D^ variant can still be modified in the presence of Mn^2+^ albeit to a lower extent, but there was also no modification in the presence of Mg^2+^. The variants GlnJ^Q42H^ and GlnJ^K85R^ show normal uridylylation in the presence of Mn^2+^ but enhanced with Mg^2+^(Figure 2A). Given the fact that only the GlnJ^Q42H^ and GlnJ^K85R^ substitutions supported modification with Mg^2+^, we combined them and constructed the GlnJ^Q42HK85R^ variant. In this case, the modification in the presence of Mn^2+^ was identical to GlnJ, but substantially improved with Mg^2+^ (Figure 2A).

**Figure 3:**
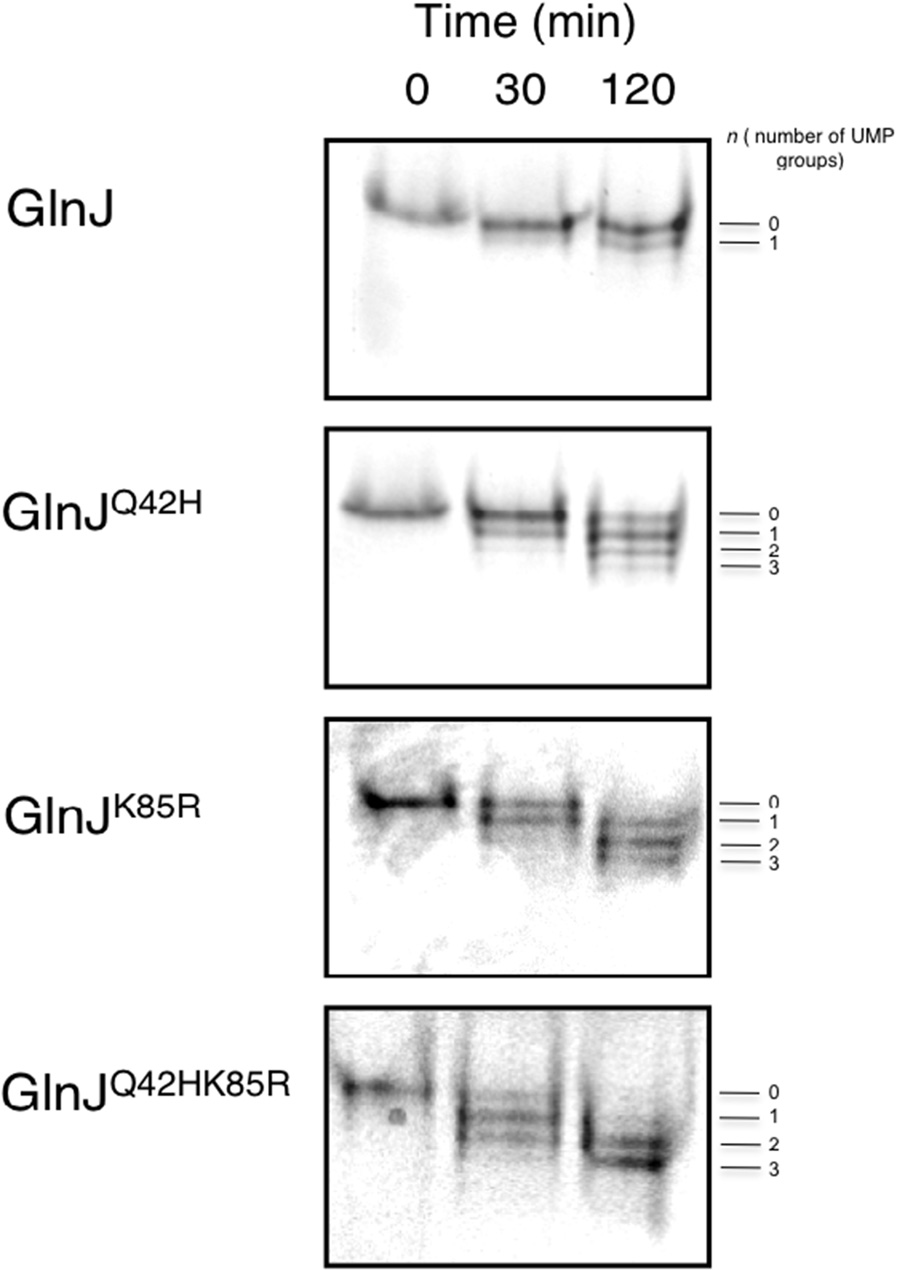
** Time-course uridylylation of GlnJ, GlnJ**^**Q42H**^**, GlnJ**^**K85R**^**and GlnJ**^**Q42HK85R**^**.** At the time points indicated samples were withdrawn and analyzed by native PAGE. The number of uridylylated subunits (0–3) is indicated.

The results shown in Figure 2 reflect the modification pattern of GlnJ and variants after 30 minutes of reaction. To better understand the modification ability of the GlnJ^Q42H^, GlnJ^K85R^ and GlnJ^Q42HK85R^ variants we performed a time-course experiment (Figure 3). On a longer time scale the modification in the presence of Mg^2+^ is even more evident in these variants when compared with GlnJ.

**Figure 4:**
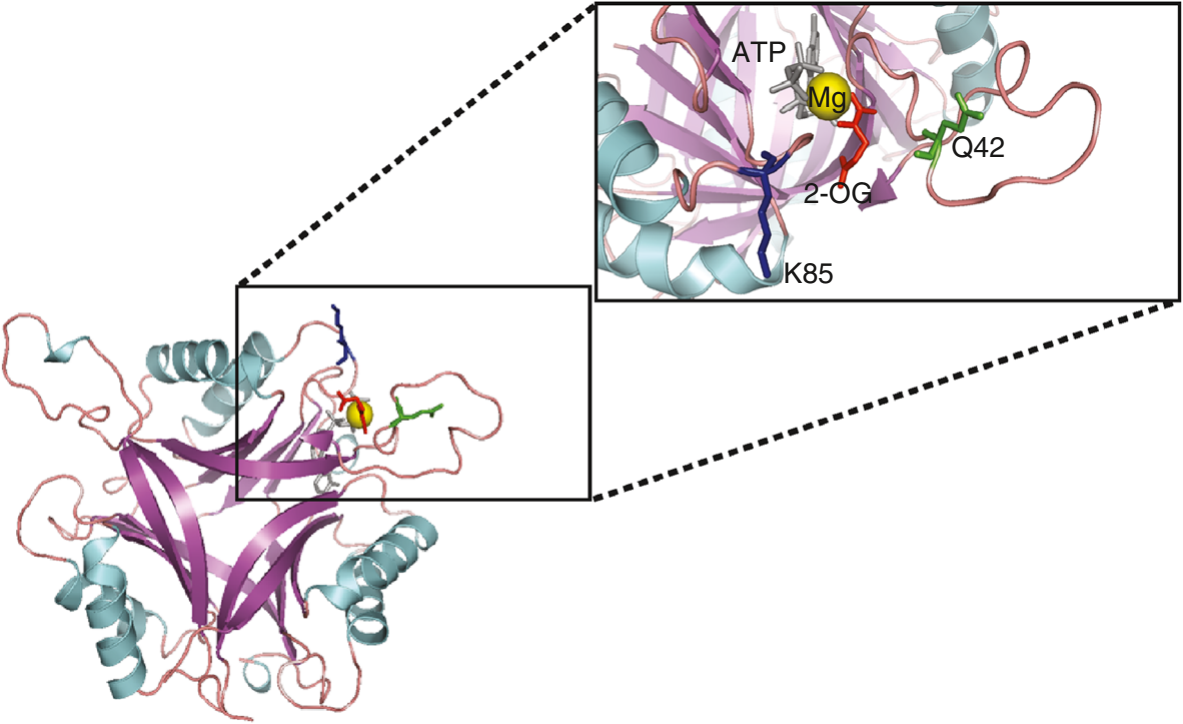
** Cartoon representation of the structural model for GlnJ, constructed based on the determined structure of *A. brasilense *GlnZ, with ligands (PDB 3MHY).** ATP is shown in gray, Magnesium ion in yellow, 2-OG in red and the residues K85 and Q42 are highlighted in blue and green respectively.

Considering the results in Figure 2A and Figure 3, it is clear that the amino acid residues at position 42 and 85 influence the activity with respect to divalent cation added in the uridylylation reaction. It could be hypothesized that these residues are either involved in the direct binding of the divalent cation or influence the architecture of its binding site in the *R. rubrum* PII proteins. Even though there is no structural information available for either GlnB or GlnJ from *R*. *rubrum*, a direct binding of the divalent cation by the residues at positions 42 and 85 is unlikely, based on the recent structural information for the homologous proteins from *A. brasilense* and *S. elongatus*[9,10]. In these structures, the residues at positions 42 and 85 are not directly involved in the coordination of the divalent cation, which occurs through the ATP phosphates, the 2-oxo acid moiety of 2-OG and the carboxamide oxygen of the Q39 side chain. Even though these residues (Q42, K85) do not participate directly in the binding of the divalent cation, they are certainly in the vicinity of the binding site, and can influence this binding by changing the conformation of the binding site or affecting binding of ATP (that could subsequently affect divalent cation binding). This is visible in the structural model of GlnJ constructed based on the structure determined for *A. brasilense* GlnZ in the presence of ligands (Figure 4). Even though a sequence identity of 74% between GlnJ and GlnZ allows the construction of a reliable model (specially for the backbone trace), the specific side chain rotamers cannot be predicted, and only a structural determination by x-ray crystallography would correctly address the influence of these two residues in the properties of the divalent cation binding site.

### GlnB variants H42Q and R85K show reduced uridylylation in the presence of Mg^2+^

Considering the influence of the residues at positions 42 and 85 we hypothesized that exchanging these residues in GlnB for the corresponding residues in GlnJ could affect Mg^2+^-dependent uridylylation. That was indeed the case, as shown in Figure 2B. The GlnB^H42Q^, GlnB^R85K^ and GlnB^H42QR85K^ variants show normal uridylylation in the presence of Mn^2+^, but that it is clearly reduced in the presence of Mg^2+^, when compared to wt GlnB.

### MnATP (but not MgATP) induces a conformational change in GlnJ

**Figure 5:**
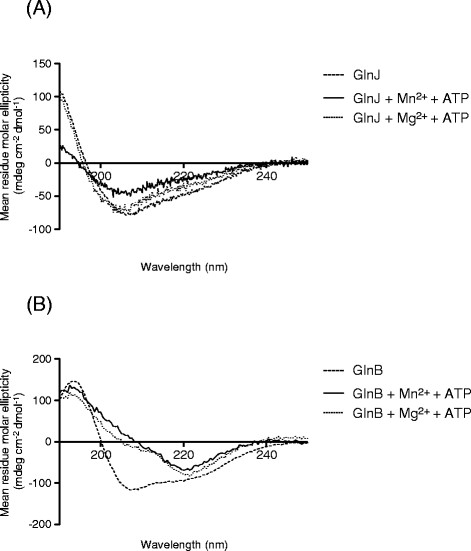
**CD spectra for GlnJ (A) and GlnB (B); protein only (dashed), protein + MnATP (solid) and protein + MgATP (dotted).** Proteins were at 100 μM trimer concentration, ATP at 10 mM and MgCl_2_/MnCl_2_ at 10 mM. Spectra were recorded at 24°C.

We hypothesized that, in the case of GlnJ, only the binding of MnATP would stabilize a protein conformation that allows the correct positioning of the T-loop for interaction with GlnD, resulting in uridylylation. To analyze this possbility we used circular dichroism (CD) spectroscopy to evaluate changes in the secondary structure of GlnJ/GlnB upon incubation with either MgATP or MnATP. It is visible from our results that only MnATP induced a conformational change in GlnJ, translated as a significant change in the CD spectrum (Figure 5A), while both Mg^2+^ and Mn^2+^ elicited a similar conformational change in GlnB (Figure 5B). These observations of divalent cation-induced conformational changes in the PII proteins correlate well with the conditions required for efficient uridylylation by GlnD.

### The GlnJ and GlnB variants retain functionality

**Figure 6:**
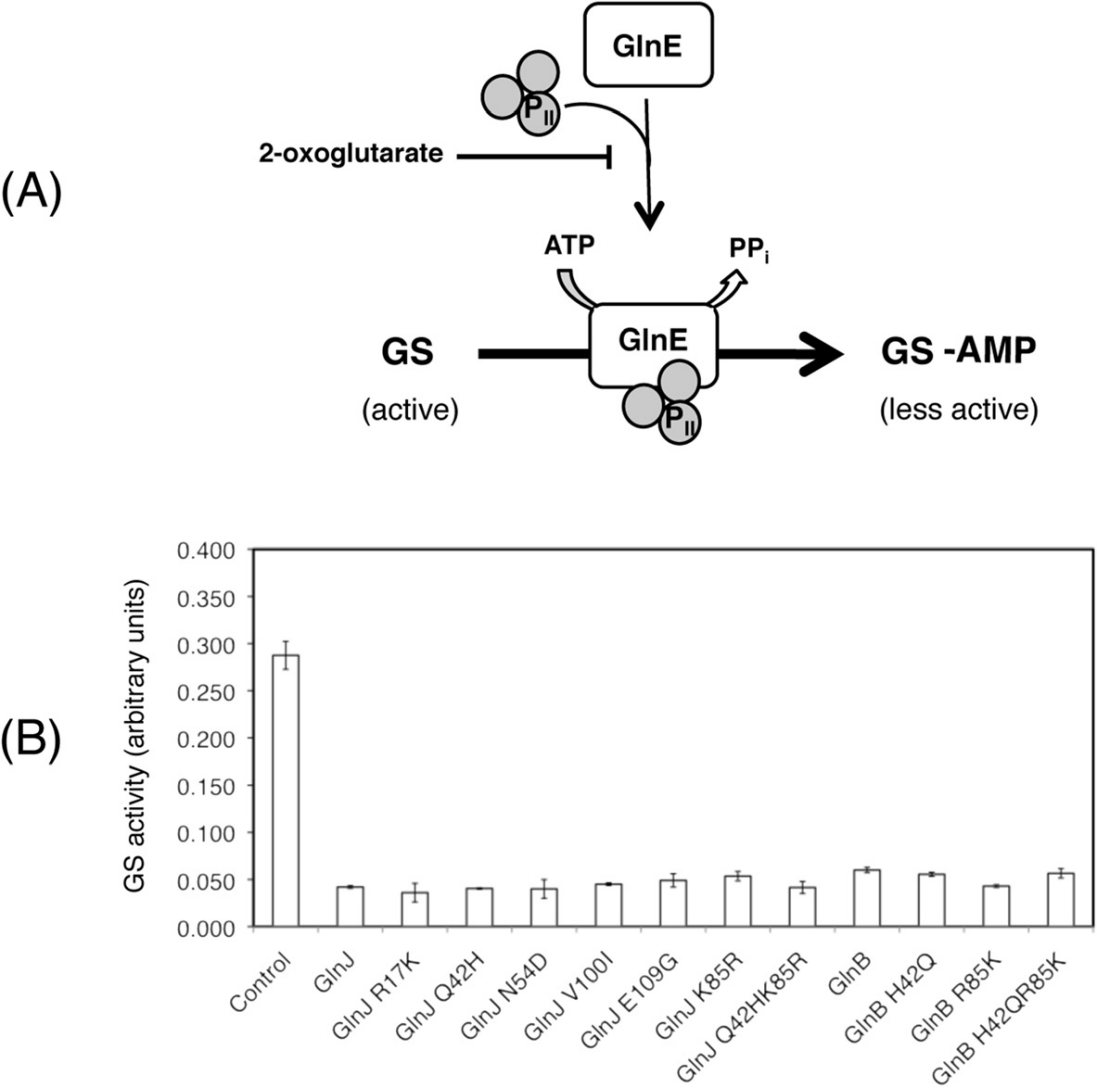
**Analysis of PII protein function in the activation of GlnE.**(**A**) Model representing the role of PII proteins in the regulation of GS activity, through GlnE in *R. rubrum *. (**B**) Glutamine synthetase activity after 30 minutes of incubation with GlnE and PII proteins (as indicated). Results are the average of three experiments and are shown as mean ± SD.

To determine if the substitutions affected protein function we analyzed the functionality of the GlnJ and GlnB variants using an assay based on one of the cellular targets of PII proteins, the adenylyltransferase GlnE. We have previously used this assay as means to determine whether PII variants are still able to perform a PII dependent function [13]. GlnE is responsible for the regulation of GS activity by post-translational adenylylation [5]. PII proteins (in the unmodified form) interact with GlnE promoting adenylylation of GS, leading to lower GS activity (Figure 6A).

To analyze the functionality of all variants constructed, we tested the ability to activate GS adenylylation by GlnE, resulting in reduced GS activity. As shown in Figure 6B, all variants tested were able to activate the adenylylation activity of GlnE.

## Conclusions

The two PII proteins GlnJ and GlnB from *R. rubrum* show different requirements in terms of divalent cations (Mg^2+^/Mn^2+^) for efficient uridylylation by GlnD. Specifically, the uridylylation of GlnJ requires the presence of Mn^2+^, with Mg^2+^ not being able to support this modification. Most likely this is due to the fact that only Mn^2+^ (or MnATP) is able to bind and induce a conformational change in GlnJ, as demonstrated here with CD spectroscopy.

We have shown that it is possible to influence the divalent cation response in the uridylylation of the PII proteins, catalyzed by GlnD, by simply exchanging two amino acid residues in the PII proteins (at positions 42 and 85, located in the T and B loops respectively). Although the substitutions constructed (Q to H and K to R) do not represent dramatic changes in the amino acid properties, these changes have a clear effect on the role of Mg^2+^ (the Mn^2+^ dependent uridylylation is retained in all variants studied). Moreover, we have also confirmed that these variants retain functionality in the GlnE-activation assay, suggesting that these substitutions do not greatly perturb the overall structure.

It is presently unclear from the structural point of view, which conformations of either GlnJ or GlnB (particularly of the T-loop) are interacting with GlnD and how these conformations are affected by the binding of different divalent cations (Mg^2+^ and Mn^2+^). Additionally, a direct translation of the present results obtained with purified proteins to an in vivo physiological situation is not linear as there is presently no information concerning the concentrations of either Mg^2+^ or Mn^2+^ in *R. rubrum*, and if these concentrations vary in response to the nitrogen status (transitions that require changes in the uridylylation of the PII proteins). Nevertheless, it is certainly possible that Mn^2+^ has an important role, as we found this divalent cation to be always required in all reactions involving GlnJ. In addition to the Mn^2+^ requirement for in vitro uridylylation of GlnJ by GlnD, we have also demonstrated that the dissociation of the GlnJ-AmtB1 complex only occurs with Mn^2+^, ATP and 2-oxoglutarate, and that Mg^2+^ can not substitute for Mn^2+^[11,13]. In addition, Mn^2+^ ions are essential for the activity of DRAG (the activating enzyme for nitrogenase) [14,17], a protein that has been suggested to interact with GlnJ [14,15]. Considering that GlnJ is only expressed under nitrogen fixing conditions [6,15], all factors that affect uridylylation of GlnJ can be of importance in the regulation of the DRAT/DRAG system and ultimately of nitrogenase.

In summary, considering that GlnJ and GlnB are remarkably similar yet retaining functional specificity, it is possible that differences in divalent cation binding and consequently in the uridylylation status of the proteins can result in different target interaction and ultimately in different physiological roles. This study adds on to the understanding of the complexity of the PII signaling system in bacteria.

## Methods

### Bacterial strains and plasmids

All plasmids and bacterial strains used in this study are listed in Table 1. *E. coli* strains were grown on selective Luria-Bertani medium containing antibiotics at the following final concentrations: 50 μg ml^-1^ ampicillin, 15 μg ml^-1^ tetracycline and 34 μg ml^-1^ chloramphenicol. *R. rubrum* S1 was grown in the medium previously described [18] under an atmosphere of 95% N_2_/ 5% CO_2_ at 30°C.

**Table 1:** Bacterial strains and plasmids used in the present study

**Strain or plasmid**	**Relevant characteristic**	**Reference or source**
Strains		
*R. rubrum*		
S1	Wild type	
*E. coli*		
BL21 (DE3) pLysS	Host for expression of PII proteins, Cm^r^	Invitrogen
BL21 Star (DE3)	Host for expression of GlnE	Invitrogen
RB9040	Δ*glnD*; host for expression of GlnD, Tc^r^	[19]
Plasmids		
pETGlnE	pET101 derivative containing *glnE*, Ap^r^	[5]
pGEXGlnD	pGEX6P-3 derivative containing *glnD*, Ap^r^	[11]
pMJET	pET15b derivative containing *glnB*, Ap^r^	[20]
pETGlnJ	pET15b derivative containing *glnJ*, Ap^r^	[5]
pETGlnJR17K	pETGlnJ derivative encoding GlnJR17K, Ap^r^	This study
pETGlnJQ42H	pETGlnJ derivative encoding GlnJQ42H, Ap^r^	This study
pETGlnJN54D	pETGlnJ derivative encoding GlnJN54D, Ap^r^	This study
pETGlnJK85R	pETGlnJ derivative encoding GlnJK85R, Ap^r^	This study
pETGlnJV100I	pETGlnJ derivative encoding GlnJV100I, Ap^r^	This study
pETGlnJE109G	pETGlnJ derivative encoding GlnJE109G, Ap^r^	This study
pETGlnJQ42HK85R	pETGlnJ derivative encoding GlnJQ42HK85R, Ap^r^	This study
pETGlnBH42Q	pMJET derivative encoding GlnBH42Q, Ap^r^	This study
pETGlnBR85K	pMJET derivative encoding GlnBR85K, Ap^r^	This study
pETGlnBH42QR85K	pMJET derivative encoding GlnBH42QR85K, Ap^r^	This study

*Ap* ampicillin; *Tc* tetracycline; *Cm* chloramphenicol.

### Site-directed mutagenesis

All GlnJ and GlnB variants were generated by standard PCR-mediated site-directed mutagenesis using the QuikChange kit (Stratagene) and according to the manufacturer’s instruction. The templates used were pETGlnJ [5] and pMJET [20].

### Purification of *R. rubrum* PII proteins

All constructs used to express PII proteins were pET15b derivatives, generating proteins with an N-terminal poly-histidine tag. All PII proteins were purified using HiTrap 1 ml columns (GE Healthcare) according to [5].

### Purification of *R. rubrum* glutamine synthetase, GlnE and GlnD proteins

GlnD was purified as a GST fusion-protein according to [11]. Glutamine synthetase was purified from wild type *R. rubrum* and GlnE was purified with a C-terminal poly-histidine tag as previously described [5].

### Uridylylation assays

Each reaction (final volume 50 μl) contained 50 mM Tris–HCl pH 7.6, 3.5 μM PII protein (GlnJ, GlnB or a variant), 0.2 μM GlnD, 100 mM KCl, 1 mM ATP, 1 mM dithiothreitol, 0.5 mM UTP and either 3 mM MnCl_2_ and 60 μM 2-OG or 25 mM MgCl_2_ and 250 μM 2-OG (in the control reactions the divalent cations were omitted and 2-OG was at 250 μM). After 30 min (or as indicated) the reaction was stopped by the addition of 5X native loading buffer (125 mM Tris–HCl pH 6.8, 50 mM EDTA, 50% glycerol, 5% sorbitol) and a 20 μl sample was loaded onto a 12.5% native PAGE prepared according to [21]. After electrophoresis the gels were stained with Coomassie brilliant blue R250.

### Adenylylation assays

Adenylylation reactions were performed as previously described [13] and GS activity measured using the γ-glutamyl transferase reaction [5,22].

### Circular dichroism spectroscopy

Far-UV CD measurements were performed on an Applied photophysics chirascan CD spectropolarimeter using a 50 μm quartz cuvette. Wavelengths in the range 190–250 nm were scanned using 0.5 nm step resolution and 100 nm/min scan speed. The spectra recorded were collected and averaged over 1–6 scans. Measurements were recorded with the temperature kept constant at 24°C using a quantum northwest TC125 temperature controller.

## Abbreviations

GS, Glutamine synthetase; 2-OG, 2-oxoglutarate.

## Competing interests

The authors declare that they have no competing interests.

## Authors’ contributions

PFT and SN designed the project; PFT and MDM performed experiments; PFT and SN wrote the paper. All authors read and approved the final manuscript.
